# Dangerous Risk Group-4 (RG-4) emergent viruses

**DOI:** 10.6026/97320630019345

**Published:** 2023-04-30

**Authors:** John T.Sinnott, Charurut Somboonwit, Sally F.Alrabaa, Paul Shapshak

**Affiliations:** 1 1Department of Internal Medicine, Morsani College of Medicine, Tampa, Florida 33606, USA

**Keywords:** World Health Organization (WHO) Risk Group 4 (RG-4) virus pathogens, NIH, NIAID, Biosafety Laboratory (BSL)-4, Arena-*viridae*, Filo-*viridae*, Flavi-*viridae*, Herpes-*viridae*, Nairo-*viridae*, Paramyxo-*viridae*, Pox-*viridae*, emergent, global warming, vector, reservoir, human, mosquito, tick, bat, pig, mammal, bird, reptile, snake, healthcare setting, biodefense

## Abstract

World Health Organization (WHO) Risk Group-4 (RG-4) pathogens are among the most dangerous of the emergent and re-emergent viruses. International health agencies, working in concert, bridge the gaps in health care for populations at risk for RG-4 viral pathogen exposure. RG-4 virus research incorporates Biodefense Program and Biosafety Laboratory (BSL)-4 technologies. RG-4 viruses include Arena-*viridae*, Filo-*viridae*, Flavi-*viridae*, Herpes-*viridae*, Nairo-*viridae*, Paramyxo-*viridae*, and Pox-*viridae*.

## Background:

**Figure 1:**
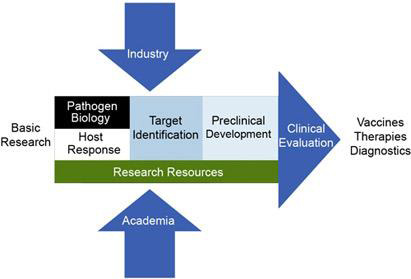
The inputs and stages of NIAID's biodefense research from basic research to the development of new diagnostics, drugs, and vaccines. Credit: NIAID, NIH. [1]

Economic, social, and ecological instability, human migration, global warming, and vector and reservoir spread, all contribute to increased numbers of emergent and re-emergent viruses, including RG-4 viruses. Very few laboratories can work with RG-4 viruses since such research is restricted to Biosafety Level 4 (BSL4) laboratories. [1] The NIH reports new Biosafety Lab facility construction projects, five NIAID-funded BSL-4 laboratory suites: the C.W. Bill Young Center for Biodefense and Emerging Infectious Diseases at the NIH (Bethesda, MD), Fort Detrick (Frederick,Maryland, NIAID Rocky Mountain Laboratories (Hamilton, Montana), Boston University (Boston, MA), and University of Texas Medical Branch (Galveston, TX). This is part of the NIAID Biodefense Program, outlined in figure 1. [1] As of 2023 there are 69 BSL4 laboratories world-wide: 15 in North America, 1 in South America, 4 in Oceania, 3 in Africa, 20 in Asia, and 26 in Europe. [2]

A few examples of emerging and re-emerging potential pandemic viruses include human immunodeficiency virus (HIV) in the1980s, severe acute respiratory syndrome (SARS)-associated coronavirus (SARS-CoV-2) in 2003, and re-emerging influenza virus pandemics in 1890, 1918, 1957, 1968, and 2010. Further, ZIKA virus in 2017, COVID-19 in 2019 and Monkey pox virus in 2022 were observed. The most dangerous viruses involve all the WHO RG-4 virus pathogens. These virus families include Arena-, Filo-, Flavi-, Herpes-, Nairo-, Paramyxo-, and Pox-*viridae*. [3, 4, 5]

## RG-4 virus families:

## Arena-viridae:

*Arena-viridae* includes Junin, Lassa Fever, Lujo Hemorrhagic Fever, Chapare, Guanarito, Machupo, Sabia, Argentinian Hemorrhagic Fever, Bolivian Hemorrhagic Fever, Venezuelan Hemorrhagic Fever, and Lymphocytic Choriomeningitis viruses. The host range and reservoirs of Arena viruses have expanded to include humans, rodents, bats, ixodid lone star ticks, fish, and reptiles (snakes). Lassa fever virus is endemic in West sub-Saharan Africa with occasional detection in the United States, Israel, the United Kingdom, Canada, Netherlands, Japan, and Germany. In endemic areas, the case fatality rate (CFR) is approximately 1-2% with an annual prevalence of 300,000 estimated infections. However, in hospitals in these areas, the CFR is closer to 69%, where infections are confirmed. Moreover, the high degree of virus genome sequence variability may contribute to variations in symptomatology. [3, 6]

## Filo-viridae:

*Filo-viridae* includes Ebola, Marburg, Sudan, Bundibugyo, Ravn, and Tai forest viruses. Of these viruses, Ebola is well-known and there was a large Ebola epidemic in West Africa, 2013-2016. The Ebola CFR ranges from 25-90%. In addition to human virus reservoirs, bats are included as well. In humans, persistent virus infections occur. Subsequent sexual transmission is observed as well, and has been seen up to 500 days after initial transmission. The virus genome undergoes significant sequence variation; thus, quantitative real-time polymerase-chain-reaction assay (qRT-PCR) is limited. To increase the reliability of qRT-PCR and minimize false negatives, two separate genome target sequences are used. Clinical diagnostic methods require improvement and vaccine improvements are also in the pharmaceutical pipeline. [3, 7, 8]

## Flavi-viridae:

*Flavi-viridae:* include Kyasanur Forest disease, Tick Borne Encephalitis, Omsk, Alkhurma, Yellow fever, Zika, Spondweni, West Nile, Yellow fever, St. Louis Encephalitis, Dengue, Japanese Encephalitis, Powassan viruses. These viruses have a wide range prevalence range, totaling 499 million, with Dengue topping the list at 390 million. Virus reservoirs include humans, mosquitoes, non-human primates, birds, pigs, rodents, lagomorphs (hares, rabbits, and pikas), and deer. The members of this virus family demonstrate a propensity for mutations in their genomes that are involved with spread among various vector mosquitoes and their hosts which likely increases their ability to infect humans. Global biomedical organizations attempt rapid tracking and containment of any viruses with the potential of becoming the next pandemic. [3, 9]

## Herpes-viridae:

*Herpes-viridae* (Herpes B virus) recently caused deaths in China. Herpes B virus, although a benign herpes virus for macaque monkeys, is very pathogenic in humans with a CFR of 70-80%. At least 50 people were infected with Herpes B virus as of 2022. Herpes B virus is also known as Herpesvirus *simiae*, Macacine herpesvirus 1, Macacine Alphaherpesvirus 1, and Cercopithecine herpesvirus 1 (CeHV-1). In 1932, the first case of human Herpes B virus was reported. The individual died of progressive encephalomyelitis 15 days after a normal monkey bit him. The virus was named Herpes B virus by Sabin in 1934. It is observed that people who work with macaque monkeys or have them as pets are at risk for Herpes B virus infection. [3, 10, 11]

## Nairo-viridae:

*Nairo-viridae* genus in the family of Bunyaviruses includes Crimea-Congo hemorrhagic fever, Ganjam, Finch creek, Nairobi sheep disease, Kupe, and Dugbe viruses. Additional sub-groups of Nairo viruses include Hughes, Der Ghazi Kahn, Qalyub, Sakhalin, and Thiafora. The geographic distribution of this virus genus ranges from sub-Saharan Africa to China and is entirely tick-borne. Crimea-Congo hemorrhagic fever, although uneventful in animal's infections, has a human CFR up to 30%. This virus is reported in the former Soviet Union, sub-Saharan Africa, Turkey, Bulgaria, Pakistan, India, China, the Arabian Peninsula, Iran, Iraq, and northern Greece. [3, 12, 13]

## Paramyxo-viridae:

The *Paramyxo-viridae* genus includes Henipa-, Respiro-, Rubula-, and Morbilli-virus groups. Hendra and Nipah viruses (Henipa group) were the first dangerous paramyxoviruses that emerged and were identified in the last century: Hendra in August of 1995 in Australia and Nipah in September 1998 in Malaysia. The related Cedar virus is not as dangerous to humans. This virus genus is zoonotic, and Hendra and Nipah human infections stem from the *Pteropus* bat species. However, these and other members of the Paramyxo-viruses are spreading to other mammals, and their phylogenetically defined clades are diverging. They are considered at risk of becoming hazardous emergent viruses. Mammals in addition to humans that are being infected include goats, cats, horses, pigs, dogs, and cows. [3, 14, 15, 16]

## Pox-viridae:

The *Pox-viridae* family includes the *Orthopox* genus, which includes Variola, cowpox, Vaccinia, and mpoxviruses. There are additional novel *Orthopox* viruses under investigation. Variola, as a human pathogen, emerged from East Africa approximately 3,000 - 4,000 years ago. Smallpox was eradicated by 1980; however, the global prevalence had reached 400 million people prior to its eradication. Mpox was first detected in primates in Denmark in 1959 and is endemic in the Democratic Republic of the Congo, Sudan, Cameroon, Central African Republic, Guinea, and Gabon. Mpox virus related disease recently emerged in clusters in non-endemic areas in what appears to be human to human transmission via close contact. Mpox virus shows neurological involvement and interacts with host immunologic response; in a way it may affect host circadian rhythms. Overall, caveats are manifest, since emergent pox viruses are most likely to continue, despite the eradication of smallpox. [3, 17, 18, 19, 20, 21]

## Conclusions:

Emergent and re-emergent of RG-4 viruses are highly pathogenic and pose profound risks for increased spread among humans, vectors, and animals. Healthcare organizations, international databases, artificial intelligence, and improved Hazmat interventions are included in the arsenals used by healthcare workers in zones where these viruses emerge. Global health and security also require monitoring RG-4 pathogen potential weaponization threats as well as corresponding countermeasures. To produce molecular mechanisms that inhibit RG-4 viruses, specific gene pathogenic mechanisms need further development. The mechanisms of inhibitions, as well as vaccines and therapeutics, could then be used as countermeasures. Further risk-benefit analyses are needed to appraise laboratories as well as open public debate about RG-4 pathogens.
